# Towards the Identification of an In Vitro Tool for Assessing the Biological Behavior of Aerosol Supplied Nanomaterials

**DOI:** 10.3390/ijerph15040563

**Published:** 2018-03-21

**Authors:** Luisana Di Cristo, Ciaran Manus Maguire, Karen Mc Quillan, Mattia Aleardi, Yuri Volkov, Dania Movia, Adriele Prina-Mello

**Affiliations:** 1 Laboratory for Biological Characterization of Advanced Materials (LBCAM) and Department of Clinical Medicine, Trinity Translational Medicine Institute (TTMI), School of Medicine, Trinity College Dublin, Dublin 8, Ireland; cmmaguir@tcd.ie (C.M.M.); karenmcquillan24@gmail.com (K.M.Q.); dmovia@tcd.ie (D.M.); 2 AMBER Centre, CRANN Institute, Trinity College Dublin, Dublin 2, Ireland; yvolkov@tcd.ie; 3 Department of Earth Sciences, University of Pisa, 56126 Pisa, Italy; mattiaaleardi@gmail.com

**Keywords:** aerosol exposure, nanotechnology-based inhalation treatments, titanium dioxide nanoparticles, air–liquid interface

## Abstract

Nanoparticles (NP)-based inhalation systems for drug delivery can be administered in liquid form, by nebulization or using pressurized metered dose inhalers, and in solid form by means of dry powder inhalers. However, NP delivery to the lungs has many challenges including the formulation instability due to particle-particle interactions and subsequent aggregation, causing poor deposition in the small distal airways and subsequent alveolar macrophages activity, which could lead to inflammation. This work aims at providing an in vitro experimental design for investigating the correlation between the physico-chemical properties of NP, and their biological behavior, when they are used as NP-based inhalation treatments, comparing two different exposure systems. By means of an aerosol drug delivery nebulizer, human lung cells cultured at air–liquid interface (ALI) were exposed to two titanium dioxide NP (NM-100 and NM-101), obtained from the JRC repository. In parallel, ALI cultures were exposed to NP suspension by direct inoculation, i.e., by adding the NP suspensions on the apical side of the cell cultures with a pipette. The formulation stability of NP, measured as hydrodynamic size distributions, the cell viability, cell monolayer integrity, cell morphology and pro-inflammatory cytokines secretion were investigated. Our results demonstrated that the formulation stability of NM-100 and NM-101 was strongly dependent on the aggregation phenomena that occur in the conditions adopted for the biological experiments. Interestingly, comparable biological data between the two exposure methods used were observed, suggesting that the conventional exposure coupled to ALI culturing conditions offers a relevant in vitro tool for assessing the correlation between the physico-chemical properties of NP and their biological behavior, when NP are used as drug delivery systems.

## 1. Introduction

The human body has a number of portals of entry available for drug administration, namely, the lungs (inhalation), the gastrointestinal tract (digestion), the circulatory system (intravenous injection), and the skin (transdermal administration) [1]. Among them, the lung, with its huge internal surface of ca. 150 m^2^, is an extremely attractive route for drug delivery, as it provides advantages for both local and systemic therapies. Firstly, pulmonary drug delivery offers local targeting for the treatment of respiratory diseases (e.g., cancer and respiratory obstructive diseases) [2], avoiding the first-pass metabolism and allowing for the direct delivery to the site of action for the treatment. As a consequence, in the future, aerosol treatments of lung cancer are envisaged to improve chemotherapy efficacy and minimize unwanted side effects [3,4]. Secondly, pulmonary administration increasingly appears to be a promising alternative for the delivery of drugs systemically [3,5,6,7,8]. In this case, however, the therapeutic efficacy of inhaled drugs is limited by their rapid clearance in the lungs [9]. Their clinical translation is also impaired by heterogeneous distribution of drug dosage, poor drug solubility in the lung fluids/mucus and induced alveolar macrophages response [10]. The development of nanoparticles (NP)-based drug delivery systems in nanomedicine offers potential effective formulation options for inhalation treatments, overcoming such limitations [11]. In particular, metal NP are an extremely promising platform for the novel treatment of neoplasms. Metal nanomaterials have a real potential to open new horizons in medicine and to pave the way for new paradigms in the next generation of theranostics [12,13].

NP systems can be delivered to the lungs in (i) liquid droplet dosage forms by nebulizers (aerosol) and pressurized metered dose inhalers, or (ii) in dry powder formulations (dry powder inhalers, DPI). However, nanoscale delivery by nebulizers is the most advanced of the three delivery platforms mentioned above [14]. As mentioned above, NP-based drug delivery systems offer the promise to reduce the shortfalls of traditional inhaled dosage forms. Nevertheless, depending on their physico-chemical properties (namely, aerodynamic diameter, shape and surface properties) [15,16,17,18], as well as on the pulmonary biological/anatomical barriers and disease state of the lungs [19], NP may, or may not, accumulate in specific regions of the human lung and interact with the cellular components of the respiratory wall [20]. Rapid clearance mechanisms (e.g., mucociliary escalator and alveolar macrophages activity) reduce for example the sustained delivery of NP-based drug delivery systems in the deep airways [3]. Thus, prior to further considering complex in vivo studies, a comprehensive in-depth knowledge of NP delivery efficiency and best formulation would be beneficial [20]. Considering this, our study was aimed at proposing an experimental design for studying the correlation among the physico-chemical properties of NP, their formulation stability, and their biological behavior. We compared two in vitro exposure methods: a widely used aerosol drug delivery nebulizer, the Aeroneb^®^ Professional Nebulizer [21,22,23], and the direct inoculation of NP suspensions, i.e., the addition of a small volume of NP suspensions on the apical side of the cell cultures by a conventional pipette. Delivery of NP by the Aeroneb^®^ Pro was chosen as subject-candidate of this study since this nebulizer is currently used in clinics and it is considered the most advanced form of NP-delivery system. This nebulizer belongs to the new generation of electronic micropump nebulizers vibrating at ultrasonic frequencies and operating continuously. It achieves a high level of efficiency in producing fine-particle and low-speed aerosol [6,24], and was demonstrated to be suitable for the delivery of novel drug delivery systems such as liposomes, nanoemulsions, niosomes, polymeric nanoparticles and PAMAM dendrimers [21,22,25]. 

Cultures of human lung (A549) cells grown at the air–liquid interface (ALI) were tested as in vitro test model. A549 cells are an accepted model for the risk assessment of inhaled NP because they mimic the alveolar epithelium, which is the primary region where most inhaled NPs deposit [26]. Currently, in vitro models of the human lung epithelium used for NP risk assessment can vary in their complexity level [26]. In this study, A549 cells were tested as mono-cultures, in an attempt to narrow down the parameters influencing the cellular responses detected and highlight the influence of the administration mode in the nanomaterials risk assessment [26]. When considering the human lung, in vitro models cultured in ALI conditions represent the more realistic screening tool currently available, considering the development and promotion of alternatives to animal experiments according to the “3Rs concept” of Russell and Burch: Replace, Reduce, and Refine [27]. Growing lung cells in ALI conditions provides in fact the physiological trait that more closely resemble the in vivo lung epithelium: it enables culturing cells in direct contact with a gas phase on the apical side, while the basolateral side is supplied with culture medium [28,29]. This knowledge represented the rationale for the selection of ALI cultures of human lung cells as the in vitro model to adopt in our study. The cell viability, cell monolayer integrity and pro-inflammatory cytokines secretion of this model were monitored as relevant biological endpoints for studying the interaction between NP and lung cells and to identify whether different administration method could modify the biological response detected.

Titanium dioxide nanoparticles (TiO_2_ NP) were selected as our case study, since they have received considerable attention as efficient drug delivery systems and interventional treatments, especially for cancer treatment [30]. For instance, based on their excellent photocatalytic activity, photo-excited TiO_2_ NP have demonstrated the capability to kill cancer cells effectively, and they can also be applied as a nucleic acid endonuclease: TiO_2_ NP-DNA nanocomposites not only retain the intrinsic photocatalytic capacity of TiO_2_ NP and the bioactivity of the oligonucleotide DNA (covalently attached to the TiO_2_ nanoparticles), but also possess the chemically and biologically unique property of a light-inducible nucleic acid endonuclease, opening new opportunities for gene therapy [31]. TiO_2_ NP have been fabricated into various shapes for drug delivery applications, such as whiskers, capsules, and porous shapes. These new drug delivery systems permit high delivery efficiency, precise control of the dose, sustained drug release and significant reduction in drugs’ side effects [30]. TiO_2_ NP have also been reported to be effective as carriers for various drugs, such as sodium phenytoin, valproic acid, temozolomide, 5-fluorouracil, daunorubicin, doxorubicin and paclitaxel [30,32,33,34,35]. In our study, we have selected to test two different TiO_2_ NP. These are the NM-100 and NM-101), two benchmark nanomaterials obtainable from the Joint Research Centre (JRC) Nanomaterials Repository. NM-100 and NM-101 have substantial differences in size and aggregation status, described in detail within our study. 

To the best of our knowledge, this is the first time where cell models grown in ALI conditions were used to compare the exposure effects of aerosolized NP to those triggered by NPs administered in suspension by pipette. In the literature several comparison studies in fact exist where the biological responses of ALI cultures to NP-aerosol exposure are compared to those detected in submerged cultures exposed through conventional methods [36,37,38,39,40]. However, submerged cultures of lung epithelium are not a representative model of in vivo exposure to inhaled nanoparticles [41].

## 2. Materials and Methods

### 2.1. Reagents

Fetal bovine serum (FBS), culture media, Bovine Serum Albumin (BSA), lipopolysaccharide (LPS, from *E. coli*, O55:B5 serotype) and Lucifer Yellow salt powder were purchased from Sigma Aldrich (Dublin, Ireland). Fisher Scientific Ireland (Dublin, Ireland) was the source of all the other chemicals, whenever not specified otherwise.

### 2.2. Titanium Dioxide Nanoparticles

Anatase TiO_2_ NP (NM-100 and NM-101) were obtained from the JRC Nanomaterials Repository (Ispra, Varese, Italy). These materials are classified as Representative Test Materials (RTM) and include a (random) sample from one industrial production batch. The sample identification number was 06841 for NM-100 and JRCNM01001a000993 for NM-101. A complete physico-chemical characterization of the TiO_2_ NP used for this study was reported in a JRC Repository Report [42] and their main properties are summarized in Table 1.

### 2.3. NP Dispersion

NPs were dispersed as a stock solution of 2.56 mg/mL by pre-wetting powder in 0.5% ethanol (96% purity) followed by dispersion in 0.05 wt % BSA-distilled water (BSA catalogue number: A9418, Sigma Aldrich). The suspension was sonicated for 16 min with a Branson 5510 sonicator bath. Few minutes prior to cell cultures exposure (nebulization or inoculation by pipetting), the NP stock suspensions were diluted in supplemented cell culture medium at the desired doses. 

### 2.4. Dynamic Light Scattering (DLS)

Measurements of hydrodynamic size distributions and dispersion stability were carried out on stock suspensions of TiO_2_ NP (2.56 mg/mL in 0.05 wt % BSA-water) and on NP dilutions in supplemented culture medium (at concentration of 25 µg/mL) before and after 24 h of incubation at 37 °C. For the analysis, 10 consecutive measurements without pause were made at 25 °C, using the Zetasizer Nano ZS (ZEN5600, Malvern Instruments, Royston, UK). 

### 2.5. Zeta Potential

Zeta potential of NM-100 and NM-101 in PBS 0.1× were evaluated using a Zetasizer Nano Z (ZEN5600, Malvern Instruments, Royston, UK). Three zeta potential measurements were taken for each sample, each made of 20 accumulations. Measurements were carried out at 25 °C and elaborated using Smoluchowski model.

### 2.6. Nanoparticle Tracking Analysis (NTA)

The average hydrodynamic radius of NM-100 and NM-101 in complex dispersion media was characterized using NTA developed by Malvern Instruments Limited (Wiltshire, UK). A NS500 instrument, equipped with a 405 nm laser in conjunction with software version NTA 3.2, was used. Solutions of NM-100 and NM-101 at stock concentrations in dH_2_O and 0.05% BSA-dH_2_O, plus NP dilutions in culture medium supplemented with 10% fetal bovine serum (FBS) at 25 µg/mL were analyzed. The BSA-dH_2_O solution and supplemented media at a dilution corresponding to that of the 25 µg/mL were also analyzed as controls. Hydrodynamic radius was measured after incubation at 37 °C for 24 h. A nanoparticles concentration that records a minimum of 200 tracks per video was undertaken to obtain statistical significance following previously validated methodology [43,44]. Six 60 s videos were recorded for each sample. Results are reported as mean, mode, and 90% distribution (D90) values ± standard error of the mean (SEM).

### 2.7. Cell Culture

A549 cell line (human adenocarcinoma cells) was obtained from the American Tissue Culture Collection (ATCC^®^) (LG Standards, Teddington, UK). The A549 cell line was authenticated using Short Tandem Repeat (STR) profiling (LGC Standards) showing that our A549 batch is an exact match for the ATCC^®^ human cell line CCL-185 (A549) (100% match between the submitted sample and the database profile). Cells were routinely cultured in a humidified atmosphere of 5% CO_2_ in air in T75 flask (Nunc, Fisher Scientific, Dublin, Ireland) in RPMI 1640 medium with GlutaMAX supplemented with 10% FBS, 1% Penicillin-Streptomycin and 25 mM HEPES. The cells were passaged every three to four days, based on flask confluence. Two days before the exposure experiments, A549 cells were seeded on the apical side of Millicell^®^ hanging cell culture Polyester (PET) inserts (1.0 µm pore size; growth area of 1.1 cm^2^) (Merck Millipore, Dublin, Ireland) at density of 40 × 10^4^ cells/ml to obtain 17,130 cells/cm^2^ (final volume: 500 μL/insert). Inserts were placed in 12-well Millipore TC-plates and 1.5 mL of medium was added to the basolateral chamber. After 48 h incubation at 37 °C under 5% CO_2_, A549 cells had reached confluence and cultures were moved into ALI conditions by changing the medium into the basolateral chamber and removing the cell medium from the apical chamber, leaving the cells exposed to the gas phase. After 24 h at 37 °C under 5% CO_2_, the cells were used for experiments. Figure S1 in the Supporting Information reports a representative image of the morphology of the ALI cultures obtained.

### 2.8. Exposure Conditions

**Figure 1:**
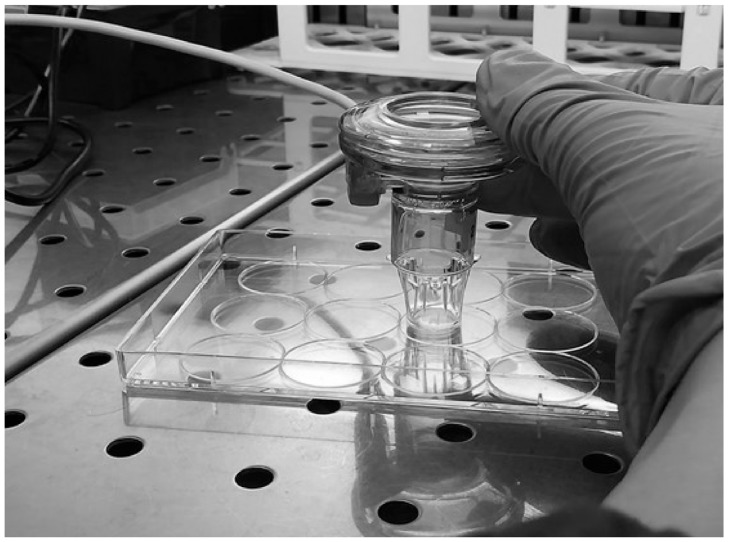
Aerosol exposure condition.

The Aeroneb^®^ Pro Nebulizer System (Aerogen, Galway, Ireland) was used to expose ALI cultures to aerosol of TiO_2_ NP. This nebulizer, which is in use in clinical settings, incorporates the OnQ aerosol generator, which consists of a membrane with ~1000 funnel-shaped apertures, in contact with a reservoir of fluid, vibrating at ultrasonic frequencies. This action extrudes fluid through the holes in the membrane, where surface tension and hydrodynamic effects result in breaking the extruded fluid into a stream of precisely controlled droplets [21]. For more information on the Aeroneb^®^ Pro nebulizer system please refer to the manufacturer website [45]. Small volume (30 µL) of NP aerosol was delivered to each ALI to mimic more closely the in vivo administration conditions. The cells were exposed for 24 h to low not-cytotoxic doses of TiO_2_ NP. These were estimated by exposing conventional submerged cultures to increasing concentrations (1, 10, 25, 50 and 100 µg/mL) of NM-100 and NM-101 for 24 h. Figure S2 in Supporting information reports the cell viability results obtained from this set of preliminary experiments. For the exposure, inserts were temporarly transferred to another plate (the exposure plate) (Figure 1). As comparison, ALI cultures were also exposed to NP suspensions by suspension inoculation, where 30 µL of NP suspension was added to the apical side of the cell cultures by a pipette. Untreated (negative) control cells were exposed directly to 30 µL of either pipetted supplemented cell culture medium or by aerosol droplets nebulization.

### 2.9. Characterization of NP Deposition Pattern Following Nebulization

Cell culture hanging PET inserts were exposed to 30 µL of NP aerosol at NP concentration equal to 1, 10 and 25 µg/mL, corresponding to the nominal concentration of 0.027, 0.27 and 0.68 µg/cm^2^ respectively, considering the area of the insert and the volume used for the exposure. A Scanning Electron Microscope (SEM) (Zeiss Ultra Plus, Zeiss, Germany) was used to evaluate the NP aerosol deposition pattern onto the inserts. A code implemented in Matlab was used to process the SEM images acquired. SEM images have been imported in Matlab and transformed into vectorial matrices.

#### Image Processing and Analysis

Image processing and quantitative measurement was carried out from the SEM images recorded, as described above. Based on the known scan area of the specimen, the area of a single pixel was derived from the total number of pixels that form a single SEM image. A threshold value was set to extract the number of pixels that could be associated with NP. Unit Pixel was associated to a particle if the corresponding value in the spot image was higher than the selected threshold (Figure S3). By multiplying the number of pixels associated to NP by the area of a single pixel, the total area occupied by NP was computed. Knowing the primary particle size (average value) and density of each nanoparticles used, available from the scientific literature for NM-100 and NM-101 [42], the dose delivered onto the cells were obtained, and expressed in µg/cm^2^. The deposition efficiency, here reported as percentage (%), was derived dividing the calculated dose deposited onto the hanging PET inserts by the nominal doses nebulized. Three separate SEM images for each concentration tested were measured and analyzed. The experiment was performed twice, and comparable results were found.

### 2.10. Characterization of Cell Responses to NM-100 and NM-101

#### 2.10.1. Resazurin Assay

To assess the viability of ALI cultures exposed to TiO_2_ NP, the resazurin assay was used, following a protocol previously published by some of the authors [46]. Briefly, after 24 h exposure to NM-100 or NM-101, A549 cells were incubated for 60 min with a fresh, serum free medium supplemented with 44 mM of resazurin, added to both basolateral and apical compartments. Fluorescence measured at 572 nm, were performed on the medium of the apical chamber by means of an FL × 800 fluorescence microplate reader (BioTek, Mason Technologies, Dublin, Ireland). Cell viability was calculated as a percentage (%) relative to the untreated (negative) control cell cultures. Since nanomaterials could interfere with this assay, a preliminary experiment was performed incubating both dyes with NM-100 and NM-101 at the highest concentration used (25 µg/mL). No fluorescence signal was detected above the background signal (data not shown).

#### 2.10.2. Lucifer Yellow Permeability Assay

The monolayer integrity of ALI cultures was evaluated following 24 h exposure to TiO_2_ NP, by means of the paracellular fluorescent marker Lucifer Yellow (LY), which absorbs at 428 nm and emits at 540 nm. To evaluate the barrier integrity of cultures, A549 cell monolayers were rinsed with Hank’s Balanced Salt Solution Buffer (HBSS) and transferred into a new 12-well plate. LY solution (0.4 mg/mL in HBSS) was added to the apical chamber of the cultures (500 μL/insert), while HBSS was added into the basolateral chambers (1.5 mL). ALI cultures were incubated for 1 h at 37 °C, after which basolateral solutions were sampled and analyzed to quantify the passage of the LY from the apical to the basolateral side. Fluorescence was measured at 538 nm by means of an FLx800 fluorescence microplate reader. To determine the concentration of LY passing through the culture/membrane, from the apical to the basolateral compartment, a calibration curve was used. The results were expressed calculating the % of LY passage, as for Equation (1):

(1) $$100\times \frac{C_{Bl}\times Bl\;volume}{C_{0}\times Ap\;volume}$$

where *C_Bl_* is the concentration of LY in the basolateral chamber, *C*_0_ is the initial concentration in the donor (apical) compartment, *Bl volume* is the volume of solution present in the basolateral chamber and *Ap* is the volume present in the apical compartment. Also, in this case we performed a preliminary experiment to test the interference of titanium dioxide nanoparticles with the LY assay. No fluorescence signal was detected above the background (data not shown).

#### 2.10.3. Cytokine Secretion

After 24 h of exposure to TiO_2_ NP, apical washes and basolateral media of the ALI cell cultures were collected and analysed for cytokines content using BioLegend ELISA kits (Medical Supply Co. Ltd., Dublin, Ireland). Tumor Necrosis Factor (TNF-α), Interkeukin-6 (IL-6), Interleukin-8 (IL-8) and Interleukin-1Beta) (IL-1β) secretion levels were quantified according to the manufacturer’s protocol. The Epoch microplate reader (Biotek, Mason Technology Ltd., Dublin, Ireland) was used to detect the optical density at 450 nm. The absorbance at 570 nm was read and subtracted from the absorbance at 450 nm to obtain the corrected (blanked) values. The cytokines concentrations were extrapolated based on a standard curve. To account for potential optical interference of NM-100 and NM-101 with the ELISA read-outs, the cytokine standards were dissolved in the assay diluent (as for manufacturer’s protocol) or in assay diluent with TiO_2_ NP-containing supplemented medium at the higher concentration tested (25 µg/mL). No significant interference with assay read-out was detected (data not shown).

#### 2.10.4. Laser Scanning Confocal Microscopy (LSCM)

After 24 h of exposure to NP, ALI cultures were washed twice with PBS and fixed with 4% paraformaldehyde (PFA) for 10 min at room temperature. Cells were permeabilized with 0.1% Triton X-100 in PBS (5 min) and incubated in blocking solution (1% BSA in PBS) at room temperature (1 h). Finally, cells were stained with Hoechst 33342 (1:1000 dilution) for nuclei and rhodamine phalloidin (1:50 dilution) for F-actin (Invitrogen, ThermoFisher, Dublin, Ireland) (room temperature; 1 h). After three washing with PBS, the filters were detached from the culture inserts with a scalpel blade and mounted on glass slides with transparent mounting medium (VECTASHIELD, Vector Laboratories Inc., Burlingame CA, USA) and imaged by LSCM. The analysis was carried out by a ZEISS 510 Meta confocal microscope equipped with a Zeiss LSM 5 software (Carl Zeiss, Oberkochen, Germany). Samples were observed through a 63× magnification oil objective lens. Qualitative confocal imaging was carried out by acquiring a series of z-stack images.

### 2.11. Statistical Analysis

One-way Anova with Bonferroni test was performed. GraphPad Prism^TM^ software version 4.00 (GraphPad Software Inc., San Diego, CA, USA) was used. Differences have been considered significant for *p* values < 0.05. All data analyzed are presented as mean values (*n_tests_* = 3) ± standard deviation and are normalized to the control untreated cells.

## 3. Results

### 3.1. Physico-Chemical Characterization of TiO_2_ NP

A detailed physico-chemical characterization of the NM-100 and NM-101, as provided in the report of JRC Repository [42], is summarized in Table 1. This characterization was integrated in our study by the measurements of surface charge, hydrodynamic size distributions and dispersion stability by Zeta Potential, DLS (Table 2) and NTA (Table 3). 

DLS measurements were carried out on stock suspensions and on NP dilutions in supplemented culture medium. Overall, DLS results showed a high hydrodynamic radius and PDI in all dispersing media for both TiO_2_ NP samples. This was probably associated with instability of NP suspensions due to agglomeration phenomena and a subsequent broad range in size distribution. Additionally, the size distribution of NM-100 and NM-101 increased drastically in FBS-supplemented media at 0 h and 24 h, suggesting further agglomeration and potentially the formation of a protein corona under the conditions adopted for biological experiments [47]. Nevertheless, in supplemented cell culture medium, NM-100 showed a more mono-dispersed population as compared to NM-101, as indicated by the PDI values. These data demonstrated that differences in the formulation stability of those two materials exist under the experimental conditions used in this study. 

Results by NTA yielded somewhat similar data to those observed using DLS. Wide size distributions were detected for both particles in dH_2_O and 0.05% BSA-dH_2_O, at stock and 25 µg/mL concentrations. In supplemented media, particle distributions appear narrow and discrete. However, overlap with the size distribution of the supplemented media at a 25 µg/mL equivalent dilution revealed that particles analyzed are likely serum protein aggregates. Figure S3 shows the size distributions of the two NP.

### 3.2. Characterization of Aerosol Delivery Efficiency

**Figure 2:**
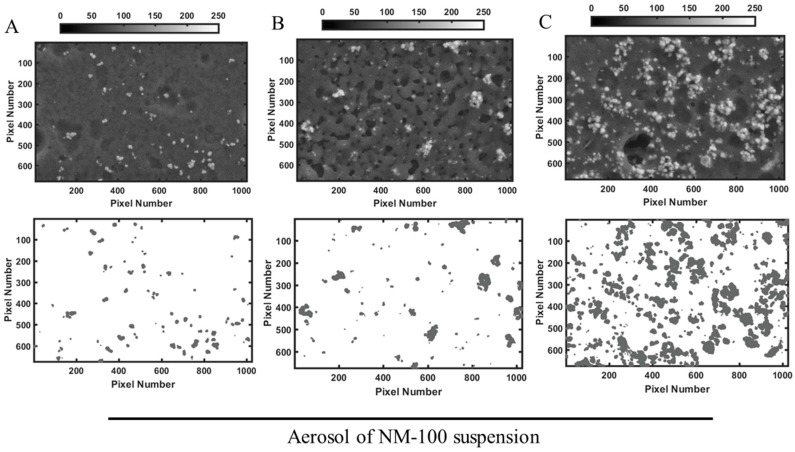
Representative images of NM-100 deposition onto PET hanging inserts. Aerosol was generated by nebulizing 30 µL of NM-100 suspension at the concentrations of (**A**) 1, (**B**) 10 and (**C**) 25 µg/mL. Inserts were imaged by SEM (upper row), and images transformed into a matrices of x rows and y columns by Matlab for analysis of deposition efficiency (lower row). Deposition of NM-100 appears homogenous. Scale bar 1 µm. Abbreviation: NM, nanomaterials.

**Figure 3:**
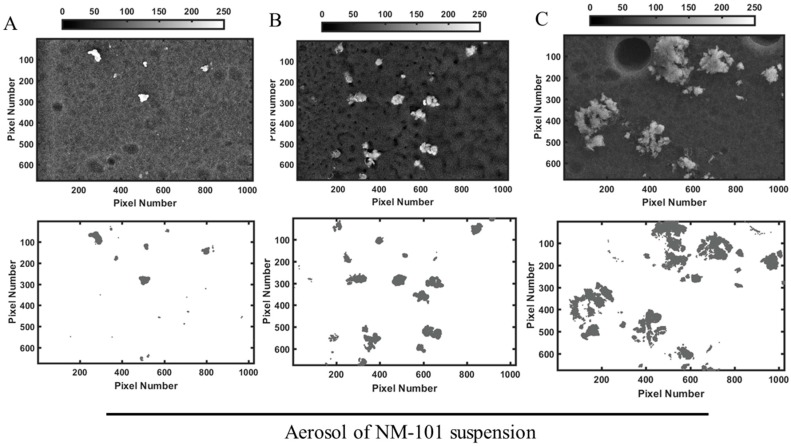
Representative images NM-101 deposition onto PET hanging inserts. Aerosol was generated by nebulizing 30 µL of NM-101 suspension at the concentrations of (**A**) 1, (**B**) 10 and (**C**) 25 µg/mL. Inserts were then imaged by SEM (upper row), and images transformed into a matrices of x rows and y columns by Matlab for analysis of deposition efficiency (lower row). Formation of small and large aggregates could be identified after the nebulization. Scale bar 1 µm. Abbreviation: NM, nanomaterials.

To characterize the aerosol generated by the Aeroneb^®^ Pro Nebulizer, NP dispersions were nebulized onto cell culture inserts in in vitro cell-free experiments. Figure 2 and Figure 3 show representative images of the deposition pattern of nebulized TiO_2_ NP and highlight substantial differences between the behaviours of the two NP tested. The deposition of NM-100 appears homogenous (Figure 1), whereas formation of small and large aggregates could be identified on the inserts when NM-101 was nebulized (Figure 2). Matlab analysis (Table 2) showed that for NM-100, the doses delivered by aerosol were very similar to the nominal concentration nebulized, with a 90% of deposition efficiency. This demonstrated the reliability of the Aeroneb^®^ Pro Nebulizer in delivering stable suspensions of inhalable TiO_2_ NP-based drug delivery systems. On the contrary, the deposition efficiency was very low for NM-101, with values below 50% as compared to the nominal concentration nebulized. We hypothesize that this result is associated with the formation of NP aggregates that remained trapped in the nebulizer mesh; this hypothesis is well supported by the DLS data (Table 2).

### 3.3. Characterization of Cell Responses to NM-100 and NM-101

**Figure 4:**
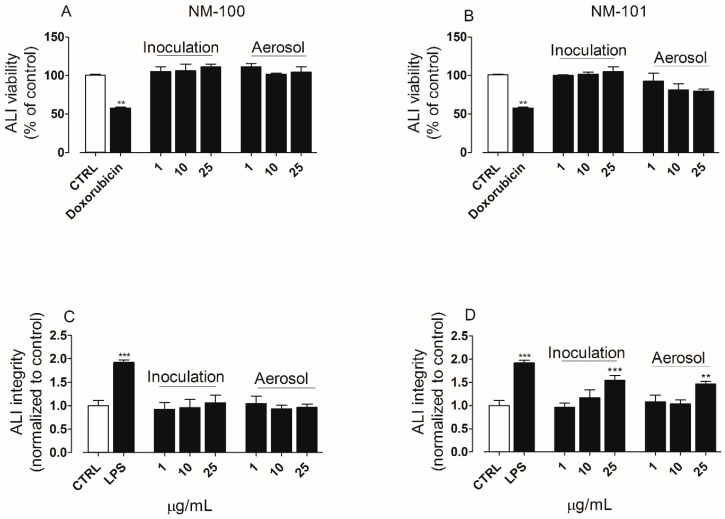
(**A**,**B**) Cell viability and (**C**,**D**) epithelial barrier integrity in air–liquid interface (ALI) cultures of A549 cells exposed to TiO_2_ NP (µg/mL) by solution inoculation or aerosol: (**A**,**C**) NM-100; (**B**,**D**) NM-101. Cell viability and epithelial barrier integrity of ALI culture were assessed by resazurin assay and LY permeability assay, respectively. Doxorubicin (20 µM, 24 h of treatment) was used as positive control for the viability assay. LPS (100 ng/mL, 24 h of treatment) was used as positive control for monolayer integrity. Untreated cultures were included as negative control (CTRL). Data are reported as mean ± standard deviation (*n_tests_* = 3). The symbols (**) and (***) indicate *p* values < 0.01 and < 0.001 vs. the untreated (negative) control (CTRL), respectively. Abbreviations: ALI, air–liquid interface; LPS, lipopolysaccharide; NM, nanomaterials.

For convenience, the concentration of the NP suspensions, expressed in µg/mL, was used to report the results on the cell responses graphs. Please refer to Table 4 for conversions to nominal dose nebulized and dose delivered (µg/cm^2^). ALI cultures were exposed by inoculation or by aerosol to NP suspensions at low not-cytotoxic doses (Figure S2). Low not-cytotoxic doses were tested in our experiments since it was already observed in different studies that significant biological effects at lower doses of particles can occur in the inserts than in the plates, in the submerged conditions [36,48]. After 24 h exposure, cell viability and cell integrity were assessed. Both TiO_2_ NP did not show cytotoxic effects (Figure 3A,B). However, a significant impairment of the epithelial barrier integrity could be detected by LY permeability assay when ALI cultures were exposed to NM-101, at the higher concentration tested (25 µg/mL, Figure 4D). No significant differences could be noted when comparing the results obtained following exposure by the two different methods adopted (solution inoculation vs. aerosol). 

**Figure 5:**
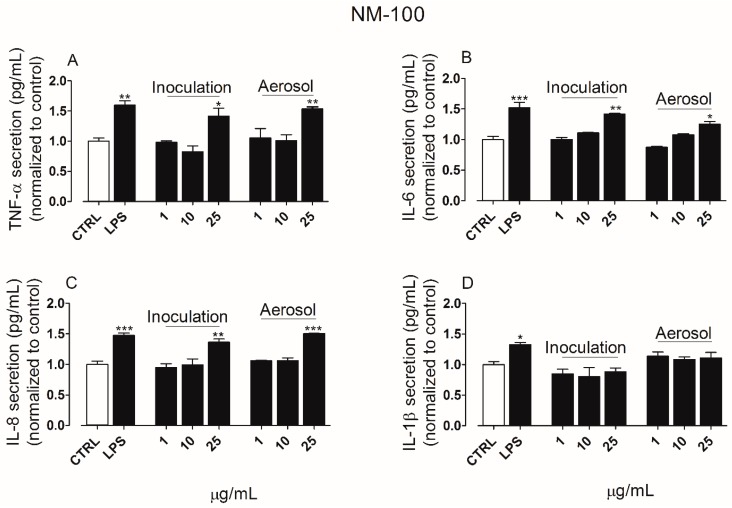
Cytokines secretion levels following exposure to NM-100 by suspension inoculation or aerosol: TNF-α (**A**), IL-6 (**B**), IL-8 (**C**) and IL-1β (**D**). LPS (100 ng/mL, 24 h of treatment) was used as positive control, while untreated cultures were included as negative control (CTRL). Data are means of three independent determinations (*n_tests_* = 3) ± standard deviation. The symbols (*) (**) and (***) indicate *p* values < 0.05, < 0.01 and < 0.001 vs. the untreated (negative) control (CTRL), respectively. Abbreviations: ALI, air–liquid interface; IL-1β, interleukin-1beta; IL-6, interleukin-6; LPS, lipopolysaccharide; NM, nanomaterials; TNF-α, tumor necrosis factor alpha.

**Figure 6:**
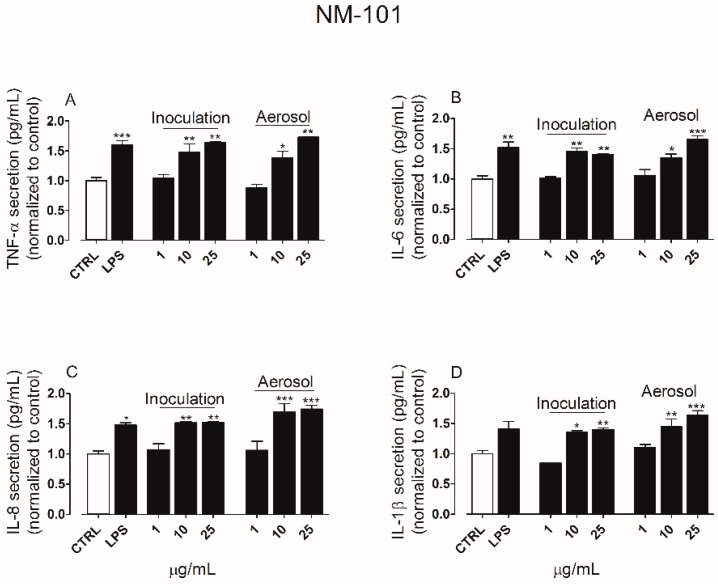
Cytokines secretion levels following exposure to NM-101 by suspension inoculation or aerosol: TNF-α (**A**), IL-6 (**B**), IL-8 (**C**) and IL-1β (**D**). LPS (100 ng/mL, 24 h of treatment) was used as positive control, while untreated cultures were included as negative control (CTRL). Data are means of three independent determinations ± standard deviation. The symbols (*) (**) and (***) indicate *p* values < 0.05, < 0.01 and < 0.001 vs. the untreated (negative) control (CTRL), respectively. Abbreviations: ALI, air–liquid interface; IL-1β, interleukin-1beta; IL-6, interleukin-6; LPS, lipopolysaccharide; NM, nanomaterials; TNF-α, tumor necrosis factor alpha.

The secretion of pro-inflammatory cytokines was quantified in the culture media derived from both apical and basolateral chambers of ALI cultures exposed to TiO_2_ NP for 24 h. In the apical side, exposure to NM-100 (Figure 5A–D) produced an increase in the secretion of TNF-α, IL-6, IL-8 but not IL-1β, whereas NM-101 (Figure 6A–D) induced a significant secretion of TNF-α, IL-6 and IL-8 even at the lower concentration tested (10 µg/mL). NM-101 caused also an increase in the secretion of IL-1β (at concentration ≥ 10 µg/mL).

No cytokines could be detected in the basolateral media (Figure S4). This result suggests that there was a polarized secretion of cytokines by the epithelial cells into the apical compartment. This has been previously reported by other research groups for various human lung cell lines (including A549 cells) grown on Transwell™ membranes [26,49,50,51]. 

**Figure 7:**
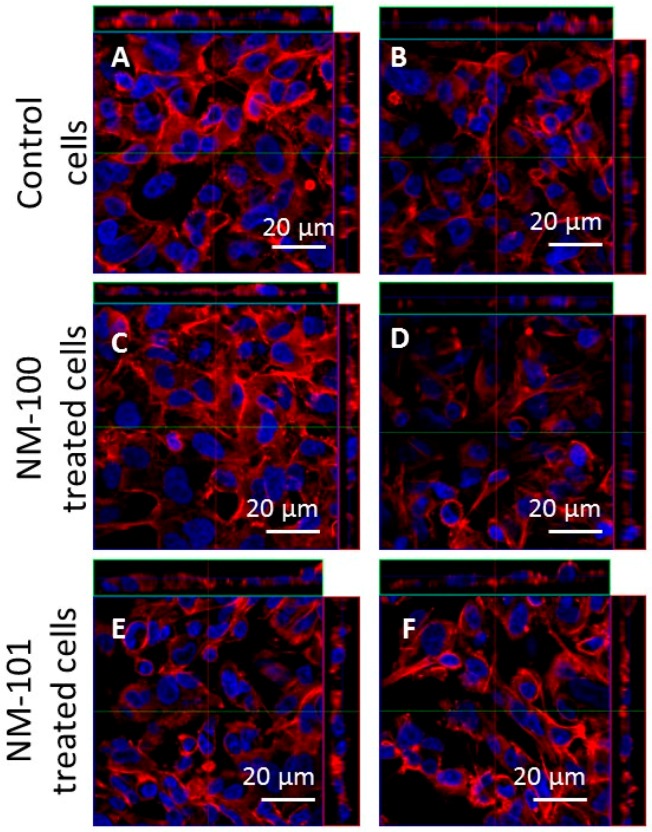
Ortho-images of representative LSCM micrographs of ALI cultures. Cells were stained with Hoechst 33342 (nuclei, in blue) and rhodamine phalloidin (F-actin, in red). Scale bars: 20 μm (63× objective lens). The left column shows the results obtained for the inoculation method; the right column shows those for the aerosol exposure method. (**A**,**B**) ALI culture control cells. (**C**,**D**) ALI cultures exposed to NM-100; (**E**,**F**) ALI cultures exposed to NM-101. Abbreviations: ALI, air–liquid interface.

No significant differences in cell responses could be found in the cell viability, epithelial barrier integrity and cytokines secretion levels when comparing the two exposure methods (inoculation vs aerosol). This result was confirmed by LSCM analysis. Indeed, the morphology of A549 cells was comparable among ALI cultures following exposure by inoculation or aerosol (Figure 7A–F). These data suggest once again that the two exposure systems used provide comparable results.

## 4. Discussion

Lungs are attractive targets for the pulmonary administration of drugs, since they offer many advantages over conventional administration [52]. Over the recent years, there has been an increased interest in aerosol therapy with advantages in drug administration including (i) a more rapid absorption into the systemic circulation, and (ii) an higher bioavailability than with other non-invasive modes of administration (e.g., oral administration) [5]. Moreover, pulmonary administration enables the delivery of low drug doses to its site of action for a localized effect (i.e., directly to airway regions), which leads to a rapid clinical response with few systemic side effects [6]. Nanoscale drug delivery systems in combination with aerosol administration hold great promise in successfully enhancing the therapeutic efficacy of inhaled drugs. However, synthesizing nanoparticles for aerosol therapy can be complicated: undefined structure/shape, poor biocompatibility, and improper surface chemistry are possible risk factors in the biological environment. A number of obstacles including immune reaction, rate of clearance from circulation, efficiency in targeting, and ability to cross biological barriers will follow when these nanoparticle systems enter the preclinical and clinical testing arenas. Identification of optimal physico-chemical parameters is critical for modulating the particle–particle interaction within a biological environment aggregation tendencies and adsorption of proteins on nanoparticle surface. A substantial variation in any of these factors can contribute to poor drug delivery, and to the loss of therapeutic efficiency and maybe to toxic events [53]. Thus, in our study, we wanted to provide an in vitro experimental design that could be used to investigate the correlation among the physico-chemical properties of NP, their formulation stability and their biological behavior at lung level, when NP are used as NP-based drug delivery systems. In this work we have chosen to test two different NP, obtained from the JRC repository of benchmark nanomaterials (NM-100 and NM-101). These two NP were selected as our case study because their physico-chemical properties have been very well characterize by JCR [42]. In fact, thanks to the knowledge of the main relevant property that distinguish these two NP, we were able to correlate these data with the results obtained from our experimental design, in term of formulation stability and biological behavior of NP. 

Aerosol deposition is influenced by several factors, including the aerosol-generating system, aerosol characteristics (particle size, shape, density, etc.) and the inhalation pattern (flow rate and volume) [6,21]. In this study we were keeping all these parameters constants (aerosol-generating system and inhalation pattern), or similar (shape, density of the particles). The only variant was the primary size of the particles and the resulting specific surface area: indeed NM-101 has a smaller size and therefore an higher surface area [42]. Our results on the characterization of aerosol deposition showed differences in the behavior of the two TiO_2_ NP similarly charged (Table 2): SEM images showed the formation of many aggregates, much bigger as the nebulized dose became higher, for NM-101 (Figure 3A–C), whereas nebulized NM-100 appeared very well dispersed (Figure 2A–C). Similarly, a high deposition efficiency was achieved only for NM-100 (Table 3). The physico-chemical properties of the two TiO_2_ NP under study can provide us an explanation of the aerosol characterization reported here. DLS results (Table 2) carried out on dH_2_O–0.05% BSA (stock solution) and on FBS-supplemented medium (condition adopted for the biological experiments), showed high values for both hydrodynamic radius and PDI in all the measurements for TiO_2_ NP samples. This was associated with the instability of NP suspensions due to aggregation phenomena. Moreover, an increase in the size distribution for both NP was registered in medium plus FBS, suggesting a further agglomeration in cell culture media, as reported by other studies [54,55,56]. However, PDI values (Table 2) have showed that NM-100 appears much more mono-dispersed in supplemented cell culture medium compare to NM-101. In addition, NTA measurements, in according with the DLS data, show significant differences in the hydrodynamic radius between NM-100 and NM-101, confirming that the particles analyzed are likely serum protein aggregates in complex dispersing medium. Thus, the differences in the formulation stability were explained by the aggregation phenomena and probably by the formation of protein corona that occurs in the condition adopted for biological experiments. The more aggregation of NM-101 suspension is strictly associated with the small size of the chosen particles. It is known that, if not prevented, particles with smaller sizes lead to a higher relative surface area and higher energy and therefore are more subject to aggregation [57]. The conclusion is that the main physico-chemical properties that distinguish NM-101 from NM-100 strongly affects the formulation stability of NP suspension, and as a consequence, the capability of by Aeroneb^®^ Pro nebulizer in delivering such NP systems. Therefore, through the investigation of the physico-chemical properties of NP and their formulation stability, we can extend this experimental approach to other nanomaterials. 

**Figure 8:**
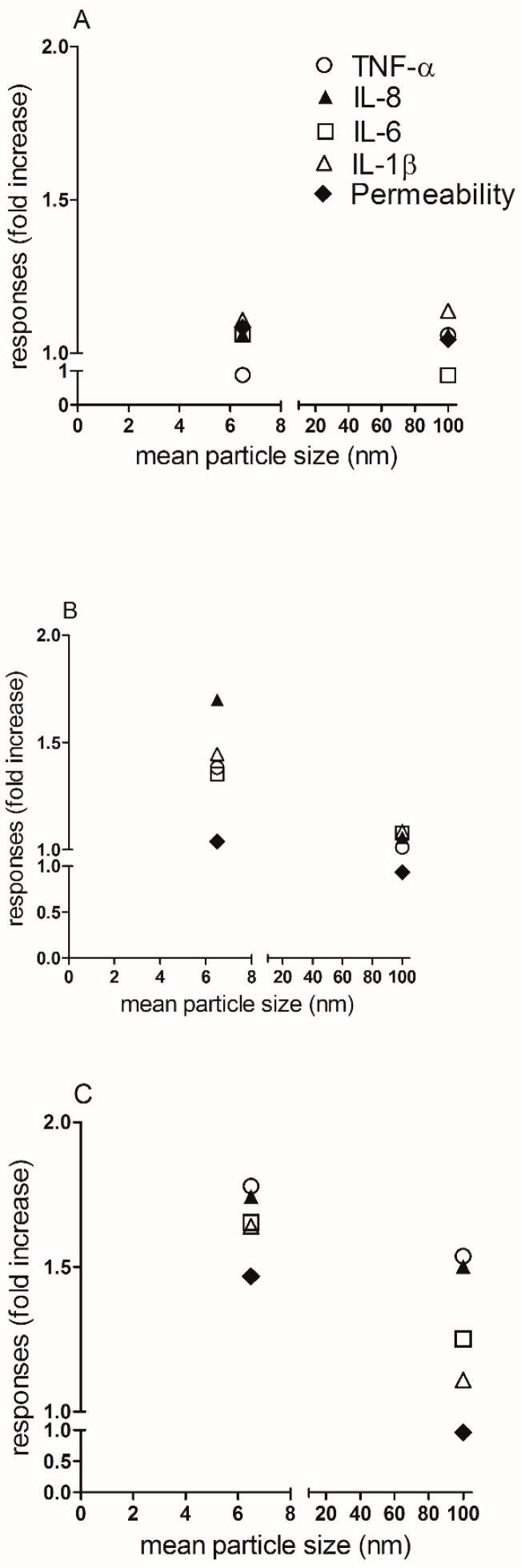
Graphical summary of the correlation between the mean particle sizes of TiO_2_ NP and their impact on cytotoxicity in ALI culture of A549 cells. In these graphs the correlation between the biological responses induced by TiO_2_ NP, under aerosol condition, and the mean particle size of NM-100 (100 nm) and NM-101 (6.5 nm), is shown. For convenience, we reported the results obtained by aerosol exposure, as we did not find any significant differences between the two exposure methods used. (**A**) 1 µg/mL of NM-100 and NM-101; (**B**) 10 µg/mL of NM-100 and NM-101; (**C**) 25 µg/mL of NM-100 and NM-101. The legend for all the three Panels is showed in Panel A. Abbreviations: IL-1β, interleukin-1beta; IL-6, interleukin-6; TNF-α, tumor necrosis factor alpha.

To understand if different exposure methods could modify the biological response to NP, ALI cultures were exposed to NP by aerosol or suspensions inoculation. We have reported comparable biological data between the two exposure methods used (Figure 4, Figure 5, Figure 6 and Figure 7). Moreover, as expected, a higher biological reactivity of NM-101 was registered when compared to NM-100, as indicated by the significant perturbation of the barrier integrity and induction of pro-inflammatory cytokines. Finally, investigating the impact of the physico chemical properties on the biological responses induced by the particles, we are able to demonstrate a correlation between the particle size (the main property that distinguish the two NP) and the biological behavior of NP. Indeed, in Figure 8 we demonstrate that with the increase of the dose of NM-101, and therefore the increase of formulation instability (Figure 3), a strong correlation between the mean particles size and the cytotoxicity can be described: as the particle size becomes smaller the cytotoxicity becomes higher.

## 5. Conclusions

Our experimental design, comparing aerosol and suspension exposure in cultures of human lung cells grown in ALI conditions, can be used for studying the correlation between the physico-chemical properties of NP, as determinants of the formulation stability, and the biological behavior of NP, when NP are used as drug delivery systems administrated by means of a nebulizer. The characterization of the aerosol deposition plus the physico-chemical characterization of the NP demonstrated that the formulation stability of NM-100 and NM-101 was strongly dependent from the aggregation phenomena occurring during the biological experiments. Hence, the differences in the formulation stability were not associated to the nebulization system used. The nebulizer adopted for the experimental tests showed once again its reliability in delivering stable suspensions of inhalable NP-based drug delivery systems; this being the case for the more stable NM-100 but not for the aggregated NM-101. Interestingly, comparable biological data were obtained between the two exposure systems used, demonstrating that the combination of the proposed methodology (ALI models + direct inoculation) represents a relevant in vitro tool for assessing the biological behavior of aerosolized NP-based drug delivery systems.

## Figures and Tables

**Table 1:** Main TiO_2_ nanoparticles (NP) physicochemical properties, as described in Joint Research Centre (JRC) Report [42].

Sample	Crystalline Phase	Primary Particle Size (nm)	Primary Density (g/cm^3^)	Specific Surface Area (m^2^/g)
NM-100	Anatase	100.0 ± 50.0	3.84	9.23
NM-101	Anatase	6.5 ± 1.5	3.84	316.07

**Table 2:** Zeta potential, dynamic light scattering (DLS) measurements and Polydispersity Index (PDI) of TiO_2_ NP. Ten consecutive measurements were collected without pause for each sample (NM-100 and NM-101). Data are shown as mean ± standard deviation (SD) (*n_tests_* = 3). For Zeta Potential three measurements were taken for each sample.

Sample	Zeta Potential (mV, in PBS 0.1×)	Dispersing Media	Concentration (mg/mL)	Time Point (h)	Z Average ± SD (nm)	PDI ± SD
NM-100		0.05% BSA-d H_2_O	2.56	0	146.9 ± 5.1	0.5 ± 0.06
	−34.0 ± 1.82	Supplemented RPMI 1640 media	2.5 × 10^−2^	0	418.2 ± 21.1	0.3 ± 0.03
				24	470.7 ± 19.6	0.3 ± 0.03
NM-101		0.05% BSA-d H_2_O	2.56	0	842.6 ± 28.7	0.3 ± 0.05
	−31.6 ± 1.46	Supplemented RPMI 1640 media	2.5 × 10^−2^	0	1663.2 ± 79.4	0.6 ± 0.08
				24	991.6 ± 72.8	0.4 ± 0.10

**Table 3:** NTA measurements of TiO_2_ NPs dispersed in DI water, 0.05% BSA-water and supplemented RPMI 1640 media. Data are shown as mean, mode, and 90% distribution (D90) values ± standard error of the mean (SEM). In addition, 0.05% BSA-dH_2_O and media controls at 2.5 × 10^−2^ mg/mL equivalent dilutions data are included as controls.

Sample	Dispersing Media	Concentration (mg/mL)	Mean Size ± SEM (nm)	Mode Size ± SEM (nm)	D90 Size ± SEM (nm)
NM-100	dH_2_O	2.56	167.4 ± 5.6	130.9 ± 16.1	280.3 ± 6.6
	0.05% BSA-dH_2_O	2.56	178.5 ± 13.0	84.6 ± 16.3	305.0 ± 25.5
		2.5 × 10^−2^	145.9 ± 19.0	112.0 ± 20.4	233.2 ± 26.1
	Supplemented RPMI 1640 media	2.5 × 10^−2^	91.6 ± 19.4	57.0 ± 15.4	151.1 ± 31.0
NM-101	dH_2_O	2.56	134.0 ± 12.5	91.2 ± 6.5	247.5 ± 37.3
	0.05% BSA-dH_2_O	2.56	136.6 ± 17.1	122.4 ± 31.9	213.1 ± 36.4
		2.5 × 10^−2^	126.3 ± 22.0	111.9 ± 18.9	177.1 ± 36.3
	Supplemented RPMI 1640 media	2.5 × 10^−2^	105.5 ± 8.2	75.6 ± 11.5	163.9 ± 13.2
0.05% BSA-dH_2_O	Particle free dH_2_O	2.5 × 10^−2^ equivalent	147.5 ± 6.3	128.6 ± 16.6	215.8 ± 10.9
Supplemented RPMI 1640 media	Particle free dH_2_O	2.5 × 10^−2^ equivalent	87.8 ± 1.9	66.2 ± 3.9	136.2 ± 3.2

**Table 4:** Characterization of NP aerosol deposition onto polyester (PET) hanging inserts in in vitro cell-free experiments. The dose delivered (expressed in µg/cm^2^) was extrapolated using a Matlab code as describes in Section 2. The deposition efficiency was calculated by diving the delivered dose by the nominal concentration of the NP suspension (expressed in µg/cm^2^). Here we reported the data obtained analyzing with Matlab the representative images reported in Figure 1 and Figure 2. The experiment was performed twice with comparable results.

Sample	Concentration of NP Suspension (µg/mL)	Nominal Dose Nebulized (µg/cm^2^)	Measured Dose Delivered (µg/cm^2^)	Deposition Efficiency (%) *
NM-100	1	0.027	0.02	92
	10	0.27	0.24	89
	25	0.68	0.58	85
NM-101	1	0.027	0.01	44
	10	0.27	0.06	24
	25	0.68	0.18	26

## Supplementary Materials

The following are available online at http://www.mdpi.com/1660-4601/15/4/563/s1, Figure S1: Immunofluorescence staining of ALI cultures. Laser Scanning Confocal Microscopy (LSCM) image of A549 cells cultured in ALI conditions, Figure S2: Assessment of viability in submerged A549 cells after exposure to NM-100 and NM-101, Figure S3: NTA size distribution, Figure S4: Representative example of the Matlab code implemented for extrapolating the deposition efficiency of NPs delivered by aerosol, Figure S5: Cytokines production by untreated cells or TiO_2_ NPs-treated cells.
