# Supplementary material for: Towards the Identification of an In Vitro Tool for Assessing the Biological Behavior of Aerosol Supplied Nanomaterials
Source: Int J Environ Res Public Health. 2018 Mar 21;15(4):563. doi: 10.3390/ijerph15040563 (PMC5923605; doi:10.3390/ijerph15040563)
Supplement: Supplementary file 1 [file ijerph-15-00563-s001.pdf]

Towards the Identification of an In Vitro Tool for Assessing the Biological Behavior of Aerosol Supplied Nanomaterials

Luisana Di Cristo [1,*], Ciaran Manus Maguire [1,2], Karen Mc Quillan [1], Mattia Aleardi [3], Yuri Volkov [2], Dania Movia [1] and Adriele Prina-Mello [1,2,*]

1 Laboratory for Biological Characterization of Advanced Materials (LBCAM) and Department of Clinical Medicine, Trinity Translational Medicine Institute (TTMI), School of Medicine, Trinity College Dublin, Dublin 8, Ireland; cmmaguir@tcd.ie (C.M.M.); karenmcquillan24@gmail.com (K.M.Q.); dmovia@tcd.ie (D.M.)
2 AMBER Centre, CRANN Institute, Trinity College Dublin, Dublin 2, Ireland; yvolkov@tcd.ie
3 Department of Earth Sciences, University of Pisa, Pisa 56126, Italy; mattiaaleardi@gmail.com
* Correspondence: dicristoluisana1987@gmail.com (L.D.C.); prinamea@tcd.ie (A.P.-M.); A.P.-M.: Tel.: +353-1-896-3259

SUPPORTING INFORMATION

Immunofluorescence staining of ALI cultures

In order to evaluate whether a confluent monolayer of cells was formed in ALI conditions, A549 cells were immunostained and specimens analysed by Laser Confocal Scanning Microscopy (LCSM) imaging. For that purpose, cells grown in ALI conditions were fixed with 3.6 % ParaFormAldehyde (PFA), 24 h after moving cells into ALI conditions, for 10 min at room temperature. Fixed cell models were incubated first with Triton-X 0.1 % (in PBS) for 3 min at room temperature for permeabilizing the cell membrane, and then with 1% BSA (in PBS) for 30 min at room temperature for blocking unspecific binding of fluorescent tags. For staining the cellular structures, cells were incubated for 30 min in the dark with Hoechst (1:1000 dilution) for nuclear staining, anti-α Tubulin Antibody-Alexa488 conjugate (1:200) for staining α-tubulin filaments, and Rhodamine Phalloidin (1:77) for staining F-actin. Finally, cells were rinsed with PBS three times, membranes detached with a scalpel blade and placed on a glass slide. Specimens were mounted with VECTASHIELD® mounting medium and sealed with nail varnish. The samples were stored in the dark at 4 °C. LCSM imaging was performed by a ZEISS 510 Meta confocal microscope, equipped with a Zeiss LSM 510 software (Carl Zeiss, Germany). A 63x/1.4NA oil immersion objective lens was used.

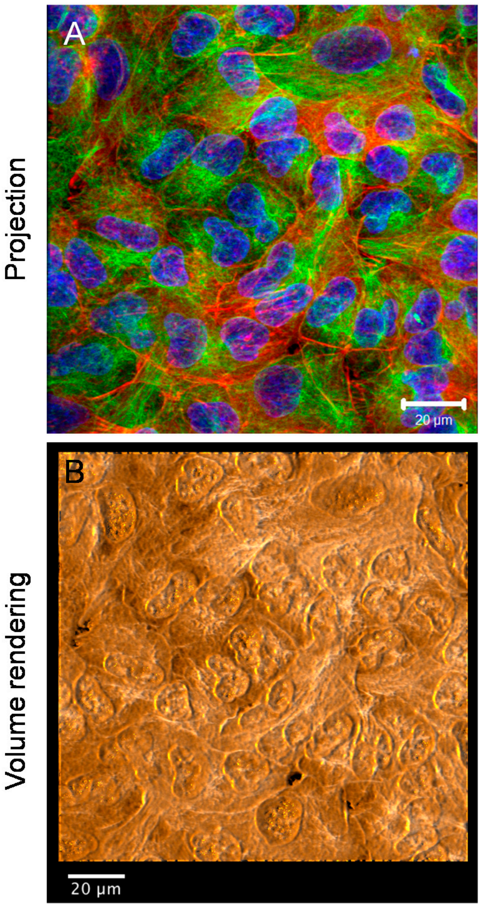

Figure S1. Immunofluorescence staining of ALI cultures. Laser Scanning Confocal Microscopy (LSCM) image of A549 cells cultured in ALI conditions. (A) Projection of A549 cells forming a monolayer. A representative sample population of cells was visualized by confocal microscopy using a 63× oil immersion lens. Nuclei were stained with Hoechst (blue), α-tubulin filaments with anti-α Tubulin Antibody-Alexa488 conjugate (green), and F-actin with Rhodamine Phalloidin (red). (B) Three-dimensional surface rendering image of the cell monolayer formed by A549 cells (in yellow as pseudo-colour) on black background. Scale bars: 20 μm.

Viability of submerged A549 cells exposed to NM-100 and NM-101

A549 cells were seeded at a density of 17,130 cells/cm^2 (200 μl/well) in 96-well plates (Nuclon Delta Surface, Thermo Scientific). Considering that the growth area is equal to 0.36 cm^2/well in 96-well plates, 6,166 cells/well were seeded. Cultures were grown under submerged culturing condition. A549 cells were treated with NM-100 or NM-101 at concentrations of 1, 10, 25, 50 and 100 μg/ml for 24 h at 37 °C. Effects of NM-100 and NM-101 on cell viability were

quantified by Resazurin assay as described under Materials and Method. Results of the experiment are shown in Figure S2. Data are shown as average ($n_{replicates}$ = 3; n_{tests} = 3) ± standard error of the mean.

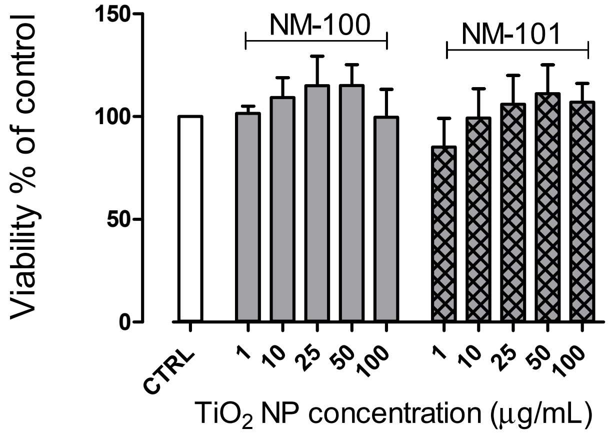

Figure S2. Viability of submerged A549 cells exposed to NM-100 and NM-101. Viability of submerged A549 cells was assessed by Resazurin assays. An untreated control (CTRL) was included in the experimental design.

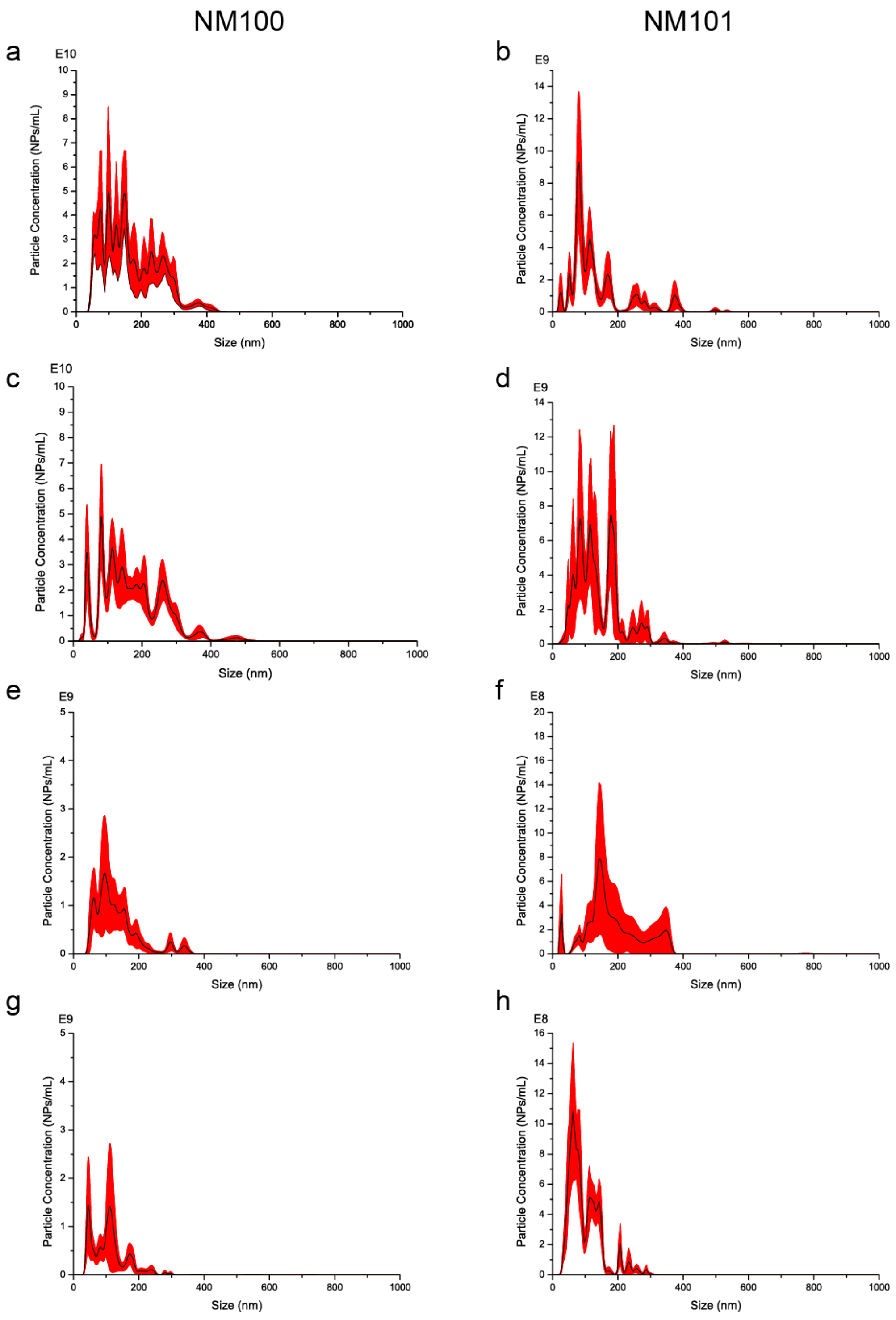

Figure S3. NTA size distribution. NTA analysis of NM100 and NM101 in dH2O (a-b), 0.05% BSA-dH2O (c-d), 0.05% BSA-dH2O at 25 μg/ml concentration (e-f), and supplemented RPMI-1640 cell culture medium at 25 μg/ml (g-h).

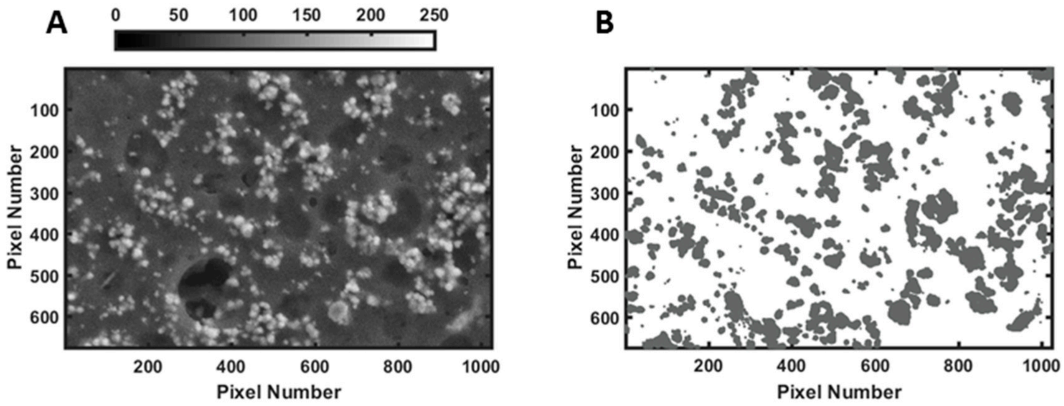

Figure S4. Representative example of the Matlab code implemented for extrapolating the deposition efficiency of NPs delivered by aerosol. (A) SEM image of the deposition of a NM-100 suspension with concentration equal to 25 μg/mL. (B) SEM image transformed into matrices of x rows and y columns by Matlab. In (B) a threshold value was set in order to extract the number of pixels associated with nanoparticles.

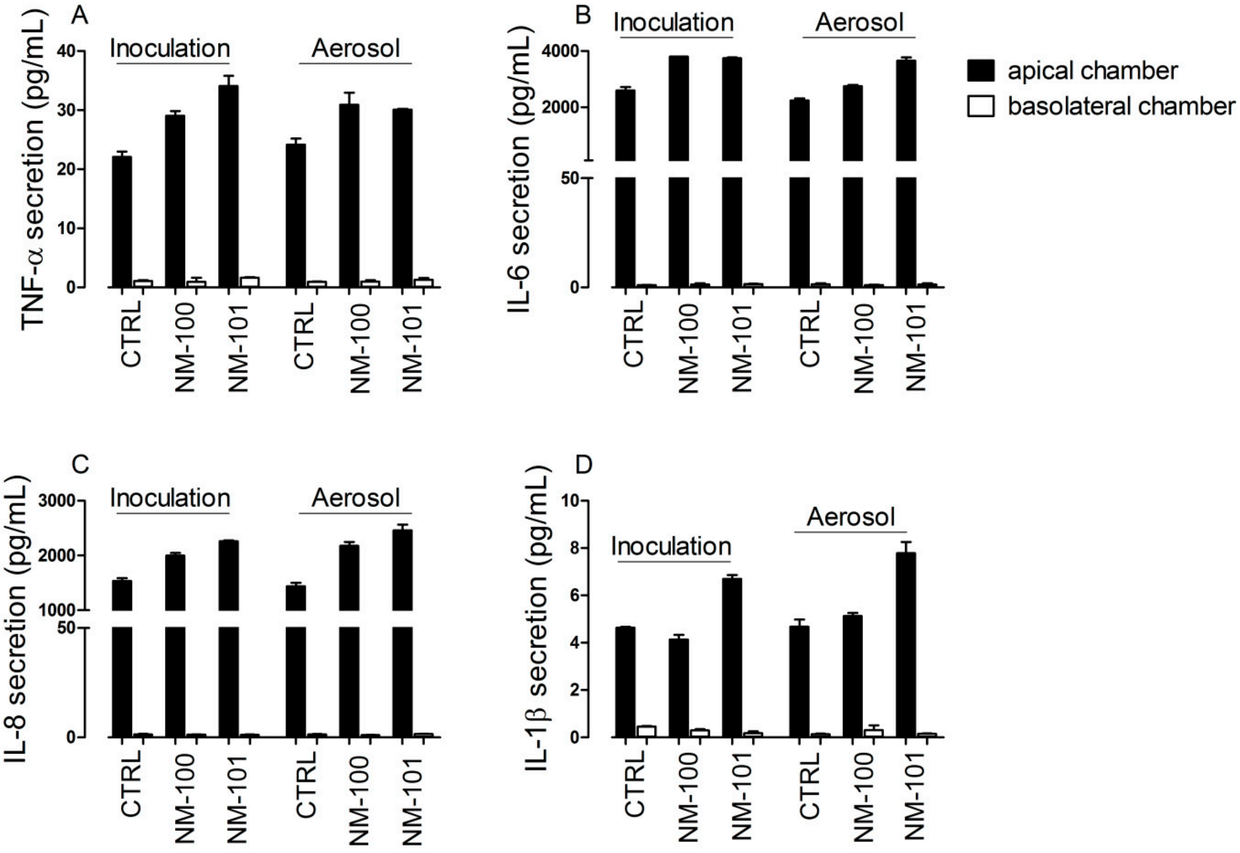

Figure S5. Cytokines production by untreated cells (CTRL) or TiO_2 NPs-treated cells (25 μg/mL). The secretion of the pro-inflammatory cytokines TNF-α, IL-6, IL-8 and IL-1β was quantified in the culture media derived from both apical and basolateral chambers of ALI cultures after 24 h exposure to both NM-100 and NM-101. A significant production of such pro-inflammatory cytokines could be detected in the apical chambers of the cultures tested, whereas no significant amount of cytokines were found in the basolateral chambers. Data are means of 3 independent determinations ± S.D.
